# Participatory Disease Surveillance Systems: Ethical Framework

**DOI:** 10.2196/12273

**Published:** 2019-05-23

**Authors:** Lester Darryl Geneviève, Andrea Martani, Tenzin Wangmo, Daniela Paolotti, Carl Koppeschaar, Charlotte Kjelsø, Caroline Guerrisi, Marco Hirsch, Olivia Woolley-Meza, Paul Lukowicz, Antoine Flahault, Bernice Simone Elger

**Affiliations:** 1 Institute for Biomedical Ethics University of Basel Basel Switzerland; 2 Institue for Scientific Interchange Foundation Torino Italy; 3 De Grote Griepmeting Science in Action BV Amsterdam Netherlands; 4 Statens Serum Institut København Denmark; 5 Sorbonne Université, Institut National de la Santé et de la Recherche Médicale, Institut Pierre Louis d’Épidémiologie et de Santé Publique Paris France; 6 German Research Center for Artificial Intelligence (DFKI) Kaiserslautern Germany; 7 ETH Zurich Swiss Federal Institute of Technology Zurich Switzerland; 8 Novartis Pharma AG Basel Switzerland; 9 Institute of Global Health, Faculty of Medicine University of Geneva Geneva Switzerland; 10 University Center of Legal Medicine University of Geneva Geneva Switzerland

**Keywords:** ethics, research, influenza, human, smartphone, public health surveillance

## Abstract

Advances in information technology are changing public health at an unprecedented rate. Participatory surveillance systems are contributing to public health by actively engaging digital (eg, Web-based) communities of volunteer citizens to report symptoms and other pertinent information on public health threats and also by empowering individuals to promptly respond to them. However, this digital model raises ethical issues on top of those inherent in traditional forms of public health surveillance. Research ethics are undergoing significant changes in the digital era where not only participants’ physical and psychological well-being but also the protection of their sensitive data have to be considered. In this paper, the digital platform of Influenzanet is used as a case study to illustrate those ethical challenges posed to participatory surveillance systems using digital platforms and mobile apps. These ethical challenges include the implementation of electronic consent, the protection of participants’ privacy, the promotion of justice, and the need for interdisciplinary capacity building of research ethics committees. On the basis of our analysis, we propose a framework to regulate and strengthen ethical approaches in the field of digital public health surveillance.

## Introduction

Advances in information technology are changing medical research [1] and public health at an unprecedented rate [2]. One of the most evident changes is that it has become easy for members of the general public to contribute to public health surveillance, practice, and policy [2] by sharing personal and health-related information through digital media. The pervasiveness of technology is underscored by the fact that as of 2018, almost 4 billion people are estimated to have access to the internet [3], and there are over 318,000 health-related mobile apps [4]. In public health surveillance (ie, public health data collection and analysis to inform public health practice [5]), vast real-time health data from informal sources (eg, health-related mobile apps and twitter) allow an early detection, prevention, and monitoring of public health threats and the potential for a prompt response from authorities to mitigate them. These informal sources have facilitated the reporting of diseases by complementing and reducing the time information is transmitted in multilevel public health infrastructures. Consequently, around the world, several early warning systems are now using this innovative approach [6].

Such activities have been termed *digital epidemiology* or *digital disease detection* [7,8]. Digital epidemiology can either be performed for nonresearch or research purposes. On the one hand, if the aims of the surveillance system are simply to monitor, control, and respond to health threats by producing data on the affected population, then this serves nonresearch purposes. On the other hand, if the purpose of the surveillance system is either to contribute to or to produce generalizable knowledge, potentially applicable to different populations and settings, then it serves research purposes [9].

Data in digital epidemiology can be obtained through 2 distinct approaches, with similar public health objectives but usually different challenges [10]. With a passive approach for data collection, data subjects are not directly informed that their everyday data (stored, eg, on social platforms, blogs, and Web search queries) are being mined and processed by advanced algorithms involved in *big data* analytics to monitor or predict disease outbreaks [10-12]. One of the first notable examples of such passive data collection approach to digital epidemiology was *Google Flu Trends* (GFT). There are numerous challenges to this approach, including *big data hubris* and unstable algorithm dynamics. *Big data hubris* states that big data are simply a replacement for data collected and analyzed by conventional means rather than an adjunct to traditional public health surveillance. Unstable algorithm dynamics refer to the continuous changes made by the company to the search algorithms, as a means of improving their searching capabilities by incorporating, for instance, new search terms. However, as the case of GFT demonstrated, these improvements led to biased estimates [13]. Nonetheless, *big data* surveillance offers an unprecedented opportunity to monitor in a timely manner the spatial and temporal evolutions of epidemics with increased granularity compared with more traditional surveillance systems, provided that the potential flaws of *big data* analytics are taken into consideration [12,13].

On the other hand, a *participatory disease surveillance* system has an active approach involving digital communities of volunteer citizens who consciously provide data. This can be done either interactively by reporting their symptoms and other relevant information through an appropriate interface or by *donating* sensor data (eg, location traces) from their digital devices. Such a participatory approach not only supports the detection of potential public health threats but also empowers individuals to reflect on them and adapt their behavior accordingly [10]. An example of such participatory disease surveillance system is the European Influenzanet Consortium, which monitors *influenza-like illness* (ILI) activity during flu seasons, with data from volunteer citizens using digital national platforms and in some cases, mobile apps [14,15]. Details on the Influenzanet Consortium and its inclusion as an adequate model to illustrate ethical issues pertinent to participatory disease surveillance systems have been covered in a previous publication [16].

In 2009, the Influenzanet Consortium was formed to standardize practices among the individual national *ILI* surveillance platforms to promote collaboration [17]. Recent research started exploring the use of crowdsourcing for detection of epidemic flu spreading, improving the self-reporting experience of symptoms with a more user-friendly mobile phone apps, and enriching the data with context data recorded by the phone’s sensors [15]. The Consortium follows the top-down model of citizen participation [2], which guarantees the scientific requirements and integrity of the disease surveillance network while relying on volunteer citizens’ data. This technology-driven public health surveillance has some benefits for its participants. Real-time information on *ILI* activity at local and national levels is provided to participants, who are also advised on strategies for disease prevention [17,18]. Importantly, the participants contribute directly to the ultimate goal of this public health initiative by providing real-time granular health-related data on *ILI* [19]. Such information complements the data of the *European Influenza Surveillance Network* (EISN) at finer levels, as EISN receives mainly epidemiological and virological data from its network of general practitioners [17,18]. The large cohorts of Influenzanet (eg, over 36,000 volunteer citizens for the flu season 2015/2016) also allow detection of even small epidemics of *ILI* [17,20]. This early detection [21] could potentially enable timely mitigation strategies to reduce the health burden of influenza and decrease health expenditures associated with increased hospitalization and treatment. In addition, Influenzanet enables research and the study of subgroups, for example, influenza vaccine effectiveness in vaccinated groups [22], attitudes toward vaccination [23], health status of population outside the health care system [24], and differences in medical care–seeking behavior across the European Union (EU) [20].

However, such top-down model of citizen participation in surveillance, specifically for research purposes, raises its own set of ethical issues on top of those inherent in traditional forms. Participants are involved actively in scientific research [2], but researchers have limited personal interaction with participants to ensure that they have indeed understood the research information provided on the national platforms or mobile apps and potential risks that their participation entails. In addition, participants could be influenced by the promise of expected benefits and the imperative of altruism. Therefore, it becomes a challenge to combine protection of research participants with the promotion of high-quality data collection for ethically acceptable research purposes [25]. In participatory disease surveillance systems, these are closely linked and mutually dependent on each other. On the one hand, ensuring participants’ trust and engagement through adequate safeguards is crucial for the sustainability and quality of these surveillance systems. If participants perceive the risk of privacy violation, they might refrain from giving important information, thus, affecting the effectiveness of disease surveillance and subsequent future public health interventions [26]. On the other hand, if surveillance systems follow low-quality standards and operate outside an ethical framework, protection and collaborations of data subjects cannot be secured. Numerous ethical frameworks have been developed in the field of public health surveillance and the use of big data [27-29]. However, a 2017 systematic review on ethical issues of public health surveillance revealed that there is a need for more context-specific analyses to guide public health practice [5]. Consequently, providing an ethical framework for the regulation of such innovative participatory surveillance methods, using a real-world example, becomes of utmost importance.

In this paper, we use Influenzanet to illustrate challenges in protection of health and other sensitive information reported in participatory disease surveillance systems. We discuss and analyze challenges and needs of participant consent in surveillance and research using participant surveillance systems data. We argue that research ethics committee (RECs) should play an important role in this developing field. Finally, we propose a framework for the regulation of digital participatory disease surveillance systems, which strengthens protection of participants’ data and privacy, while promoting the concept of justice.

## Consent

### Traditional Informed Consent in Internet-Based Surveillance

In public health surveillance, there are 2 antithetical forces. Although these systems pursue the improvement of population health through surveillance of diseases (such as in the case of Influenzanet, providing protection for vulnerable populations at risk of serious adverse outcomes of influenza infection), they must also safeguard individuals against any abuse of their data by researchers [30,31]. To strike a balance between the pursuit of societal welfare and protection of individual rights, consent from participants plays a fundamental role. Originating from the necessity to protect research subjects both physically and mentally, written informed consent is traditionally obtained for medical research, and its importance has emerged even more in the current era of data protection [32]. However, this type of consent seems to be poorly adapted to the collection and use of digital data in public health surveillance [2].

In light of the inadequacy of traditional informed consent for participatory public health surveillance, 1 potential response is to reject the need for further consent in these types of studies [33] because of the fact that participants enroll on their own and not in continued medical contact. The “no problem” solution rests also on the assumption that consent is not necessary, as in public health surveillance, individual interests may be put aside to protect the public good [33]. For example, in the United States, the issue of consent in public health surveillance is circumvented by considering the latter as public health practice instead of research, thus exempting it from institutional review boards’ approval [34] and in most cases, of traditional informed consent requirements. Indeed, a participatory disease surveillance platform active in the United States, *Flu Near You*, received a waiver for informed consent [35]. However, this approach may not be the best solution for participatory disease surveillance where data are actively generated by participants, underscoring the urgent need to adapt the traditional model of informed consent more adequately to this type of surveillance system.

Informed consent in research was originally designed for studies involving a limited number of participants where it was practically and financially feasible for researchers to engage participants, provide details about the research, and obtain written informed consent before the beginning of the study [32]. A further problem with traditional informed consent is that it was designed to authorize the use of data only by those subjects and for those purposes according to which the data had been originally collected. It was, however, not intended to also cover retrospective research on samples or data. In the case of *big data* for surveillance of infectious diseases, which is often retrospective in nature (2 out of the 3 electronic data sources are medical encounter and nonhealth digital data) [12], obtaining traditional informed consent proves problematic, as it requires disclosing all potential risks of primary and retrospective research, but the latter are usually unknown at the time when data are collected [32].

There have been substantial efforts made by some Influenzanet national platforms at their outset to ensure some form of personal interaction with their participants to better explain the nature of the surveillance system. In 2003, the original Belgian/Dutch platform, called *de Grote Griepmeting* (ie, the Great Influenza Survey), received a lot of media attention, which led to the registration of tens of thousands of participants in 2003/2004. The participants’ age distribution from youth to the elderly and their wide geographic spread and different levels of education made de Grote Griepmeting more accurate and quicker to signal the onset of a flu epidemic than the general practitioners’ surveillance system organized by Netherlands Institute for Health Services Research (NIVEL). The Belgian/Dutch research team invited participants for an information, question and answer session, where they were provided with notes on the rationale of the survey questions to the flu survey study. Moreover, a forum was also created where participants could ask any remaining questions, and when specific virology questions were asked, consultation would follow with partners from the NIVEL and the National Institute for Health and Environment in the Netherlands. The team managed to answer all incoming questions from participants by email and during various local, national, and regional live radio interviews from people listening in. In 2009, the team started a public community on Facebook named *De Grote Griepmeting–Influenzanet*, where members would have their questions answered by the team. Such measures (ie, the information session and the team answering all additional questions received via email, through their forum, on the social platform Facebook, and during radio interviews) could be viewed as an equivalent solution to obtaining the informed consent of these participants (De Grote Griepmeting, email communication, April 3, 2018 and February 7, 2019).

The inadequacies of traditional informed consent have led to the development of many other ethically acceptable solutions. For instance, in retrospective research where risks are minimal, consent would not be necessary as long as the right to opt out and the right to be forgotten are preserved and enforced [36,37]. Alternatively, the requirement of informed consent upholds but is paired with waivers, which dispense researchers from requesting consent for secondary use of data, if the recontacting and reconsenting are unfeasible or would lead to nonrepresentative samples [38].

Another alternative to traditional informed consent is an *extended* version of consent, which is more suitable to public health/*big data* research, known as broad or general consent [2,32,39-41]. The key difference between traditional and broad or general consent is that data subjects provide their consent for entire classes of research [42]. This extended form of consent differs from blanket consent as data subjects do not give permission for any use of their data but rather define in broad terms the purposes of use [42]. Moreover, broad consent is only considered acceptable if 2 criteria are met. First, every new study needs to be approved by an REC or another competent entity [43]. Second, the right of participants to withdraw their consent at any time has to be maintained [32,44]. Despite the presence of these safeguards, consensus on whether broad consent can be considered truly informed is lacking [45-47]. The informative nature of broad consent rests on the assumption that autonomy is protected, as REC approval is necessary, and strategies to regularly update the data donor on ongoing opt-out opportunities are devised [48]. Furthermore, any modification to the research should automatically lead to reconsenting procedures [49]. However, broad consent cannot be entirely informed because of the unspecified nature of future research [48]. Although broad consent seems suitable for secondary uses in public health research involving digital communities of volunteer citizens or *big data*, it is uncertain whether broad consent represents the best solution in terms of respect for autonomy. Given the issues raised by broad consent and the fact that it requires initial face-to-face contact, seeking consent electronically could be an ethically satisfying alternative to traditional informed consent.

### Electronic Consent, An Adaptation of Traditional Informed Consent

Electronic consent (e-consent) implies that participants give informed consent using an information technology (eg, digital technologies). In this sense, e-consent does not represent a new form of consent but simply an adaptation of informed consent to the electronic environment [50]. E-consent is currently being used in the Influenzanet Consortium and in similar participatory surveillance platforms such as *Flu Tracking* (Australia and New Zealand) as a valid form of consent for participants. Data subjects agree to the conditions, terms, and privacy policies when registering on their respective national platforms [16,51].

Although e-consent offers the substantial benefit of a tailored fit to the digital environment, it also has some inherent problems. A unique feature of internet-based research is the absence of personal interaction between researchers and participants, where researchers would traditionally be able to provide individually tailored information and answer any question participants might ask concerning the study and the collection of health data. Therefore, one of the major risks posed by e-consent is the provision of consent through automatic processes in the digital world, as parties are not directly involved. The provision of consent is rather based on a set of computer rules determining whether access to an individual’s data by researchers could be granted on reasonable grounds [50]. For this reason, it is possible that participants provide their consent without fully understanding—or even reading—the information, terms, and conditions that data collectors provide by simply clicking the relevant buttons in the digital forms [52]. We thus recommend that several precautions ought to be implemented when e-consent is obtained. For instance, e-consent should be designed in such a way that information is delivered through a simple PDF file where participants digitally put their signature (instead of clicking a button) to increase the likelihood of the document being read. Alternatively, other possibilities offered by information technology could be exploited to help verify participants’ understanding of the information provided during the e-consent process. These include tools such as the use of audio files, PowerPoint presentations, videos, pictures, or gamification (for instance, through quizzes and animation) [53].

Though the above recommendations could foster the informed nature of e-consent, the lack of personal interaction between study participants and researchers remains. Therefore, a properly implemented e-consent would be particularly beneficial in those studies where it is impossible to provide individual counseling and where the conditions, terms, and privacy policies would otherwise not be read [54,55]. In addition, one might even argue that participants are potentially less likely to consent under undue influence or constraints because of limited interaction with researchers. They can thus easily decline consent by signing out from the digital platforms whenever they feel the need to do so [52]. In this regard, e-consent increases the autonomy of participants.

Nonetheless, a further challenge raised by the absence of face-to-face contact is how to ensure that participants have the required legal capacity to legitimately give their e-consent after they electronically authenticate [52], because of the difficulty of verifying the participants’ identity. In this sense, even if measures were taken—such as quizzes or questionnaires—to ensure that participants have understood the research information, there would be little guarantee that those quizzes or study questionnaires are actually being completed by data subjects. A potential solution to this authentication issue could come from advances made in biometric identification technologies, commonly used for security purposes [52]. For instance, the use of face recognition technology [56] on computers and mobile phones or fingerprint recognition sensor in smartphones (eg, Touch ID by Apple Inc) could be used to verify the identity of participants throughout the process of e-consent. However, processing biometric data raises additional ethical and legal issues, in particular with respect to privacy. Biometric data, similar to genetic material, carries biological traits that are unique to data subjects and which could be easily used to reidentify them [57]. However, it must be noted that, although entailing sensitive personal information, the processing of biometric data can be lawful even without subjects’ consent if processing serves the public interest or scientific or statistical research purposes (eg, Article 9.2.(j) of the European General Data Protection Regulation, GDPR) [58]. Ethical concerns with respect to biometric data might also be mitigated if, for example, the gathered biometric information was stored locally on the participants’ computers or mobile devices and not transmitted to the research team or any third party.

In the case of multinational studies such as Influenzanet, an adequately implemented e-consent could consist of a standard informed consent information form [16], as a single PDF file, being delivered to participants at the time of their registration on the digital platform and followed by a new document each and every time new information is added on the single country websites. The information provided would have to be reader-friendly and succinctly summarized, thus nudging participants to read it thoroughly. Awareness of all potential ramifications because of their participation could be further improved through the provision of quiz questions. Grading of these quiz questions could then serve as a proxy to ensure adequate understanding of the informed consent information. This method has been employed at the *Harvard Personal Genome Project* (PGP) [59]. Participants were even provided with a study guide and were required to pass an enrollment test to be considered for the project. This additional burden to participation, which is justified for genetic research (*genetic exceptionalism*), should nonetheless remain minimal for Influenzanet to retain engagement of its participants. This is supported by the mildly sensitive nature of the gathered information and the low risks associated with this kind of surveillance. Indeed, the enrollment examination for the PGP was the main barrier to participation, with almost 60% of its users dropping out [59]. Digital signature of the consent form could also be a more personalized alternative, and it would also provide additional evidence on the identity of the participant, which altogether would enhance the informed nature of this e-consent procedure.

## Protection of Subjects’ Privacy

Epidemics forecasting studies and other public health research often gather useful and sensitive data on their participants, potentially interfering with their privacy. In the case of Influenzanet, protection of participants’ privacy is secured by data anonymization and the use of a centralized database [18].

One might argue that full anonymization is not necessary for some public health surveillance, as part of the collected data is only mildly sensitive (eg, age group and gender) and thus poses only a minor threat to the fundamental rights and the privacy of participants even in case of misuse [60]. However, even nonpersonal information could be used to reveal much more sensitive information on data subjects if the former is coupled with additional geographical information, which is often collected by public health surveillance systems [61]. For instance, 1 of the core functions of Influenzanet is to map cases of *ILI* for the identification of hotspots of influenza outbreaks to model disease progression and implement effective prevention strategies. This spatiotemporal dimension of collected health data can enhance the privacy-invasive nature of epidemics forecasting research such as Influenzanet [62]. The collection of sensor and usage data from smartphones adds additional behavioral and context information, which, as shown in related work [15], has the potential to improve forecasting and risk analysis. Despite these potential benefits, even apparently nonpersonal data, such as a list of installed apps, can be an additional risk to the participant’s privacy [63]. The Consortium took great care in protecting the privacy of its participants. In case of sensor data, information is processed directly on the user’s device and only transmitted to Influenzanet in anonymized and highly aggregated form [15]. Location information of reported cases is, for example, never mapped to the individual level but rather to the postal code level [17], with only the aggregate number of cases shown. Some platforms went even further by randomizing virtual locations around the center of a large number of postal code areas taken together (eg, the De Grote Griepmeting platform). The grouping of postal codes areas was paramount for better protection of the privacy of participants, for example, in the case of a single participant in a postal code area (De Grote Griepmeting, email communication, April 3, 2018).

However, with increasing technological capabilities to integrate and analyze health data with local data, there are risks of leakage of sensitive information concerning participants’ locations, which may lead to stigmatization of the particular locations as well as residents [62,64]. Even with full anonymization, cross-referencing of essential data gathered for epidemics research purposes (eg, sex, age, and medical conditions) with other databases could eventually lead to reidentification of data subjects [2,65]. For instance, 2 researchers showed it was feasible to reidentify individuals by matching a deidentified database on *Netflix* movie recommendations to available Web-based information (eg, Internet Movie Database) [66]. Hence, anonymization per se is not a sufficient measure to adequately protect privacy. The long life span of some anonymized datasets, which is often the case with epidemics forecasting studies, de facto increases the risks of reidentification and privacy breaches through repetitive data enrichment over time [54]. Consequently, reidentification should be considered a real risk for data subjects [2] even in case of anonymization. It is, thus, paramount to ensure ethical and accountable sharing of anonymized datasets between research institutions and to combine anonymization with other adequate data security measures to prevent misuse of data and unauthorized reidentification.

## Justice

The concept of justice in research ethics is fully embodied by the policies of the Influenzanet Consortium as participation is free, open, voluntary, and nondiscriminative of any resident of the respective countries (except for Sweden, where some representativeness and comparison purposes are guaranteed by allowing participation through invitations only) [17]. This ensures a fair distribution of risks and benefits to all research participants and the public at large. If epidemics forecasting studies keep up with the high standards in terms of justice (ie, participation to the surveillance system is free, open, voluntary, and nondiscriminative) followed by the Influenzanet Consortium and similar platforms such as Flu Near You [35], the only remaining challenge would be dealing with those limitations on participation that are inherent to digital technologies. These limitations are commonly referred in the literature as the “ *digital divide* ” [2,67], and they concern both access to and proficiency with digital technologies. Access to digital technologies is also a product of many sociodemographic elements such as age, educational level of participants, ethnic groups, and their socioeconomic status [68,69]. This is reflected in the data collected by Influenzanet, which present an underrepresentation of younger age and elderly groups, an overrepresentation of the middle age group for both genders, and a higher educational level of participants in comparison with the general population [70]. In this respect, it could be claimed that epidemics forecasting studies such as Influenzanet are potentially empowering a more dynamic, informed societal group with a penchant for digital technology, whereas at the same time perpetuating the health inequalities between others [2]. However, it must also be stressed that public health surveillance benefits the public at large and not exclusively the participants. Public health surveillance, like biomedical research, is a public good, as the health benefits resulting from its interventions (based on knowledge generated from data subjects) are ultimately going to be shared with society [71]. In addition, the digital divide is decreasing annually, with technology becoming more and more pervasive [72]. Nevertheless, concerns about justice can be avoided only if results and disease prevention strategies are shared evenly and on a regular basis among all societal groups, something which the Influenzanet Consortium is promoting (eg, weekly national surveillance bulletins, regular press releases during the study, and radio broadcasting) [73,74]. Such regular results dissemination initiatives undeniably help in ensuring that expected benefits of research are shared more equally between societal groups. This could be further improved by granting access to more targeted and granular information on influenza activity to nonparticipants [74] under the concept of solidarity [75]. Such measures would allow a better protection of society as the spread of an influenza epidemic is an individual as well as a collective concern.

## Capacity Building for Research Ethics Committees

Influenzanet and similar systems are faced with multifocal ethical and legal issues. For the safeguard of data subjects, appropriate oversight and specific regulation might be needed in the future. Currently, such oversight is beyond the governance capacity of RECs, as technological advances outpace national regulatory frameworks and undermine the definitions of those concepts—such as “anonymization,” “encryption,” and “personally identifiable information” [55]—upon which RECs rely. However, RECs should be actively involved in the design and implementation of public health research involving digital communities of volunteer citizens or big data. These RECs need to act as safety nets to fill the gaps of the current regulatory framework, which often dates back to an era where modern computational and technological capabilities were not foreseeable [55]. In this perspective, we recommend RECs to undergo interdisciplinary capacity building in those innovative research methods through mutual exchange of information and training with citizen science experts, big data researchers, data scientists, ethicists, legal experts, and sociologists. This would allow the identification of ethical and legal grey zones. Stakeholders could further anticipate potential conflicting situations resulting from the enactment of new legislation. This appears even more urgent as we have entered the GDPR era. This regulation came into force in May 2018, to replace the EU Data Protection Directive 95/46/EC [1,76]. The GDPR tries to harmonize EU data protection laws with the goal of guaranteeing the same level of freedom and protection to EU citizens, while protecting personal data during cross-border sharing with international organizations and third countries [76]. This legislative reform is likely to have a considerable impact on consent requirements and exemptions from obtaining consent [1]. This could affect the expected benefits that *big data* can bring to society by increasing the regulatory burden on public health surveillance studies [1]. Furthermore, as stressed in the study by Mittelstadt, the GDPR classifies “data concerning health” as a “special category of personal data” [77]. As this category includes any personal data that reveals information on the health status (physical or mental) of participants [77], health-related information gathered from Influenzanet participants or similar epidemics forecasting studies might—until properly anonymized—fall in this special category. It is, thus, possible that detailed limitations to health data usage are imposed in the future because of the protective stance endorsed by the GDPR [77]. Therefore, interdisciplinary capacity building and acquaintance of RECs with this innovative and developing research field will be paramount to proactively ensure an adequate protection of data subjects while preventing the development of additional research barriers. Such barriers could undermine the excellent contribution to the preservation of public health made by epidemics forecasting systems such as Influenzanet.

## Ethical Framework for the Regulation of Participatory Disease Surveillance Systems

We propose the following 4 components ethical framework to provide guidance on how to ensure an adequate ethical oversight of participatory disease surveillance systems while safeguarding participants’ privacy and eliminating barriers to the work of these surveillance platforms (Table 1).

**Table 1:** Ethical framework for the regulation of participatory disease surveillance systems.

Principle	Ethical component	Considerations
Autonomy of participants	Electronic consent	Standard, reader-friendly, and multilingual informed consent form with succinctly summarized information (eg, as a single PDF file) delivered at the time of registration and each time new information is added to the digital platforms
		Informed nature of consent can be fostered through the provision of a few quiz questions to reduce the risk of participants simply “clicking through” the consent process
		Require digital signature of the consent form to incentivize participants to read the information form and as evidence of their identity
		Making participants aware of the fact that despite best effort to protect their privacy, the residual risk of a privacy leak cannot be ruled out
Nonmaleficence	Protection of participants’ privacy	Anonymization of participants’ data should be combined with other data security measures such as a highly protected centralized database for storage of participants’ data
		Location data of participants should never be mapped to the individual level but rather to the postal code level to reduce the risk of reidentification in case of rare value entries
		Sensor data from mobile phones should only be transmitted in anonymized and highly aggregated form
		Ensure ethical and accountable sharing of anonymized datasets between research institutions to reduce reidentification risks for participants through database triangulation
Justice	Access to information on disease activity and prevention strategies	Free, open, and nondiscriminative participation should be offered to members of the general public
		Disease prevention strategies and results obtained through the participatory surveillance platforms should be disseminated on a regular basis to members of the public through various means
Beneficence and nonmaleficence	Research ethics committees (RECs)	Interdisciplinary capacity building of RECs is required to keep up with technological advances, thereby ensuring an adequate protection of data subjects
		RECs should play a proactive role in the design and implementation of public health research involving digital communities of volunteer citizens
		RECs should act as safety nets to prevent barriers to public health surveillance by identifying ethico-legal grey zones and anticipate potential conflicting situations resulting from the evolving legal landscape

### Conclusions

In the developing field of participatory disease surveillance systems, the main ethical dilemma is how to ensure adequate protection of data subjects while at the same time obtaining the full benefits that public health surveillance directly involving digital communities of citizens could bring. In this complex situation, 1 of the key ethical safeguards proposed in our framework is a properly implemented e-consent. To pursue this objective, national platforms of the Influenzanet Consortium will put continuous effort in enhancing and adequately developing their e-consent procedures. Current e-consent procedures could be improved by providing standard, reader-friendly, multilingual information about the study, participants’ rights, the risks associated with their participation, and, in addition, a short series of quiz questions to verify proper understanding of the potential benefits and risks. Furthermore, requiring participants to digitally sign the Web-based consent form could both serve as a motivation for them to read properly the information provided and as a solution to allow personal identification. However, such additional burdens of participation need to remain minimal to ensure the sustainability of the platforms.

## Abbreviations

e-consent: electronic consent
EISN: European Influenza Surveillance Network
EU: European Union
GDPR: General Data Protection Regulation
GFT: Google Flu Trends
ILI: influenza-like illness
NIVEL: Netherlands Institute for Health Services Research
PGP: Personal Genome Project
REC: research ethics committee
